# Refractory Rickets

**DOI:** 10.1007/s12098-023-04538-4

**Published:** 2023-04-19

**Authors:** Amish Chinoy, Raja Padidela

**Affiliations:** 1 Department of Paediatric Endocrinology, Royal Manchester Children’s Hospital, Manchester, M13 9WL UK; 2 Faculty of Biology Medicine and Health, University of Manchester, Manchester, M13 9PL UK

**Keywords:** Phosphate homeostasis, Vitamin D-dependent rickets, X-linked hypophosphatemic rickets, FGF23, PTH

## Abstract

Nutritional rickets, caused by vitamin D and/or calcium deficiency is by far the most common cause of rickets. In resource-limited settings, it is therefore not uncommon to treat rickets with vitamin D and calcium. If rickets fails to heal and/or if there is a family history of rickets, then refractory rickets should be considered as a differential diagnosis. Chronic low serum phosphate is the pathological hallmark of all forms of rickets as its low concentration in extracellular space leads to the failure of apoptosis of hypertrophic chondrocytes leading to defective mineralisation of the growth plate. Parathyroid hormone (PTH) and fibroblast growth factor 23 (FGF23) control serum phosphate concentration by facilitating the excretion of phosphate in the urine through their action on the proximal renal tubules. An increase in PTH, as seen in nutritional rickets and genetic disorders of vitamin D-dependent rickets (VDDRs), leads to chronic low serum phosphate, causing rickets. Genetic conditions leading to an increase in FGF23 concentration cause chronic low serum phosphate concentration and rickets. Genetic conditions and syndromes associated with proximal renal tubulopathies can also lead to chronic low serum phosphate concentration by excess phosphate leak in urine, causing rickets.

In this review, authors discuss an approach to the differential diagnosis and management of refractory rickets.

## Introduction

Rickets describes a deficiency in the mineralisation of bone at the growth plate. The common pathway to all forms of rickets is a low serum phosphate, either directly due to renal phosphate wasting (hypophosphatemic rickets) or secondary to hyperparathyroidism (in cases of calcium or vitamin D-related rickets). The low serum phosphate impairs the apoptosis of terminally differentiated chondrocytes in the growth plate, which results in the presenting features of rickets [1].

By far the commonest cause of rickets is nutritional rickets, due to dietary deficiency in calcium and/or vitamin D intake. Therefore, it would be common practice, particularly in resource-deprived settings, to treat cases of rickets presenting clinically empirically with oral calcium and vitamin D supplementation. However, if despite adequate compliance to these regimes, rickets persists, then ‘refractory’ causes of rickets must be considered. These can be subdivided into those that affect phosphate metabolism (hypophosphatemic rickets) and those that affect vitamin D metabolism (vitamin D-dependent rickets). Indeed, if biochemical testing for rickets has been undertaken prior to treatment for nutritional rickets, then clues to the small proportion of cases of refractory rickets can be established (Table 1). This review article describes the current and up-to-date knowledge of these refractory forms of rickets.

**Table 1:** Biochemical features of nutritional rickets, hypophosphatemic rickets and vitamin D-dependent rickets

Test	Nutritional rickets	Hypophosphatemic rickets	Vitamin D-dependent rickets
Phosphate	Low	Low	Low
25-hydroxyvitamin D	Very low/low	Low/normal	Variable
Parathyroid hormone	High/very high	**Normal**	High/very high
Corrected calcium	Low/normal	Normal	**Low/very low**
Alkaline phosphatase	High	High	High
1,25-dihydroxyvitamin D	High	Normal/high	**Very high/low**

Parameters in bold highlight the key biochemical differences that would make one consider hypophosphatemic rickets or vitamin D-dependent rickets

## Hypophosphatemic Rickets

### Phosphate Homeostasis and Metabolism

The skeletal system stores 90% of body phosphate; outside the skeleton almost all of the phosphate is intracellular. Phosphate is therefore predominantly an intracellular ion and most of its physiological function and structural role is within the cells [2].

The bones store most of the body’s phosphate and its extracellular function is predominantly in bones where (a) it forms part of hydroxyapatite crystal (the mineral component of the bones) and (b) facilitates the mineralisation of the growth plate by causing apoptosis of the hypertrophic chondrocytes [1].

Most of the hormones required for calcium and phosphate homeostasis are common such as calcitriol [1,25,(OH)_2_D], parathyroid hormone (PTH) and fibroblast growth factor 23 (FGF23).

Phosphate absorption from the gut is either passive, across the concentration gradient, through the paracellular route while active absorption is transcellular and occurs through sodium-phosphate cotransporter 2b (NPT2b), the expression of which is controlled by 1,25(OH)_2_D. Since most diets are rich in phosphate, passive absorption sufficiently absorbs the required phosphate and active absorption is seldom required. Retrieval of phosphate from the glomerular filtrate is mediated through sodium-phosphate cotransporter 2a and 2c (NPT2a and NPT2c) located in the luminal surface of the cells of proximal renal tubules. PTH and FGF23 facilitate the excretion of phosphate in the urine by reducing the expression of NPT2a and NPT2c.

### Forms of Hypophosphatemic Rickets

Low serum phosphate concentration leads to failure of its extracellular function of mineralisation of osteoid tissue and apoptosis of hypertrophic chondrocytes causing osteomalacia and rickets respectively. Dietary phosphate deficiency causing rickets is unheard of as most diets are rich in phosphate; all forms of phosphopenic rickets are therefore secondary to excess loss of phosphate in urine either mediated through excess FGF23 or proximal renal tubular disorders (inherited genetic disorders or drug-induced).

High FGF23-related hypophosphatemic rickets is caused by genetic disorders which are mentioned in Table 2. Non-FGF23–mediated renal tubular dysfunction and associated hypophosphatemia are also mentioned in Table 2.

**Table 2:** Conditions associated with hypophosphatemia

Disorder (Genes involved where applicable)	Clinical features
*ADHR (*FGF23*)	Milder phenotype with symptoms presenting in adolescents and adults with osteomalacia
*ARHR 1 (*DMP1*)	Similar to XLH (see below) with skeletal and dental phenotypes
*ARHR 2 (*ENPP1*)	Two clinically distinct phenotypes. Around half of the infants develop lethal generalised arterial calcification of infancy and the survivors progress to develop ARHR 2
*ARHR 3 [Raine syndrome] (*FAM20C*)	A lethal condition of congenital sclerosing osteomalacia with cerebral calcification. FGF23 levels are elevated and cause ARHR 3 in some of the survivors
*XLH (*PHEX*)	Rickets in weight-bearing joints, osteomalacia, lower limb deformities (genu varum/valgum), dental abscess, short stature and craniosynostosis
*MAS/FD (Somatic mutations in *GNAS*)	Varying phenotypes which may include combinations of Café-au-lait spots, fibrous dysplasia lesions in bones, precocious puberty, phosphaturia causing rickets, osteomalacia and fragility fractures, thyroid abnormalities, growth hormone excess and Cushing syndrome
*Linear nevus syndrome (Somatic mutations in *KRAS, HRAS* and *NRAS*)	A multisystem disorder characterized by sebaceous nevus and other abnormalities outside the skin, affecting the brain, eyes and bones
*Tumor induced osteomalacia	A rare paraneoplastic syndrome associated with high FGF23 causing phosphaturia, osteomalacia and profound muscle weakness
*Iron infusion in iron deficiency anaemia	Intravenous elemental iron especially in the form of ferric carboxymaltose increases intact FGF23 concentration causing a reduction of serum phosphate
^**$**^Hereditary hypophosphataemic rickets with hypercalciuria (*SLC34A3*)	Reduced renal sodium-phosphate cotransporter 2c, reduced phosphate reabsorption, severe rickets and osteomalacia, short stature, nephrocalcinosis and renal stones
^**$**^Fanconi renotubular syndrome 2 (*SLC34A1*)	Reduced renal sodium-phosphate cotransporter 2a, causing proximal renal tubular damage and generalised renal losses of phosphate, glucose, bicarbonate, amino acids, and other solutes
^**$**^Dent disease – 1 & 2 *CLCN5* (Dent disease 1) or *OCRL1* (Dent disease 2). X-linked recessive disorder	Proximal renal tubular dysfunction characterized by proteinuria, hypercalciuria, and at least one additional finding including nephrocalcinosis, nephrolithiasis, hematuria, hypophosphatemia and chronic kidney disease
^**$**^Nephropathic cystinosis (*CTNS*)	Cystinosis commonly presents in early infancy with Fanconi syndrome, and if left untreated, leads to kidney failure
^**$**^Hereditary tyrosinemia (*FAH*)	Failure to thrive, developmental delay, cirrhosis of liver and Fanconi syndrome
^**$**^Fanconi-Bickel syndrome (*SLC2A2*)	Glycogen accumulation in the hepatic and renal system leading to severe renal tubular dysfunction and impaired glucose and galactose metabolism
^**$**^Drug-induced. For e.g., sodium valproate, heavy metals antibiotics, antiretroviral and anticancer medications	History of drug intake associated with either selective phosphaturia or generalised renal tubular acidosis, rickets, osteomalacia and fragility fractures

*Conditions associated with high FGF23 concentration. ^**$**^Conditions associated with the tubular leak of phosphate and low FGF23 concentration

*ADHR* Autosomal dominant hypophosphatemic rickets, *ARHR* Autosomal recessive hypophosphatemic rickets, *MAS/FD* McCune-Albright syndrome and Fibrous dysplasia, *XLH* X-linked hypophosphatemic rickets

### Approach to Diagnosis and Management of Hypophosphatemic Rickets

In comparison to nutritional rickets, phosphopenic rickets is rare and therefore, in the absence of family history, it is not uncommon for most of the cases to be initially diagnosed and managed as nutritional rickets.

X-linked hypophosphatemic (XLH) rickets is the most common form of phosphopenic rickets with an incidence of approximately 1 in 20–25,000 individuals [3]. It is an X-linked dominant disorder caused by a pathogenic variant in the *PHLEX* gene which increases the concentration of circulating intact FGF23. XLH becomes clinically apparent in late infancy or early childhood when rickets develops in weight-bearing joints. Most children present with rickets, waddling gait and bowed legs which are not responsive to standard treatment for nutritional rickets. Muscle pain and weakness are less prominent than nutritional rickets. Short stature is universal in children unless treatment is commenced in early infancy in those who have a family history of XLH. Some children present in mid/late childhood with genu valgum and, in isolation or combinations of symptoms of, short stature, multiple dental abscesses and craniosynostosis.

A family history of XLH helps in early diagnosis and treatment. Infants with XLH can have normal serum phosphate for 6 mo or more; therefore, biochemical screening in early infancy is generally not reliable in confirming the diagnosis and hence genetic testing should be undertaken to confirm the diagnosis.

Clinical features of all the others forms of hypophosphatemic rickets are summarised in Table 2.

### Diagnosis of Hypophosphatemic Rickets

The biochemical hallmark of hypophosphatemic rickets is low serum phosphate, elevated alkaline phosphatase, low TmP/GFR and normal or marginally elevated PTH.

Serum phosphate and alkaline phosphatase concentrations vary with age with higher concentrations in infants and children; adult values are reached after the completion of puberty. Hence age-appropriate reference ranges should be used [4]. Serum alkaline phosphatase serves as an important biomarker of the activity of rickets and for monitoring treatment [5].

All forms of phosphopenic rickets are caused by renal loss of phosphate and in children measurement of TmP/GFR using the equation: TmP/GFR = serum phosphate – [(urine phosphate × serum creatinine)/urine creatinine] allows assessment of renal capacity to reabsorb phosphate [6]. TmP/GFR essentially represents the theoretical lower limit of serum phosphate below which all filtered phosphate would be reabsorbed. Age-adjusted normal ranges are available for TmP/GFR and as a general rule at any age, it is similar to the normal serum phosphate ranges for the age [7].

Genetic testing to confirm the pathogenic variants casing hypophosphatemic rickets is the gold standard in confirming the diagnosis. Gene panel is more cost-effective compared to single gene testing and should be undertaken if finance and facilities are available. Genetic testing allows confirmation of diagnosis, firm assurance on management protocol to follow and genetic counseling for prognosis and future pregnancies.

**Fig. 1:**
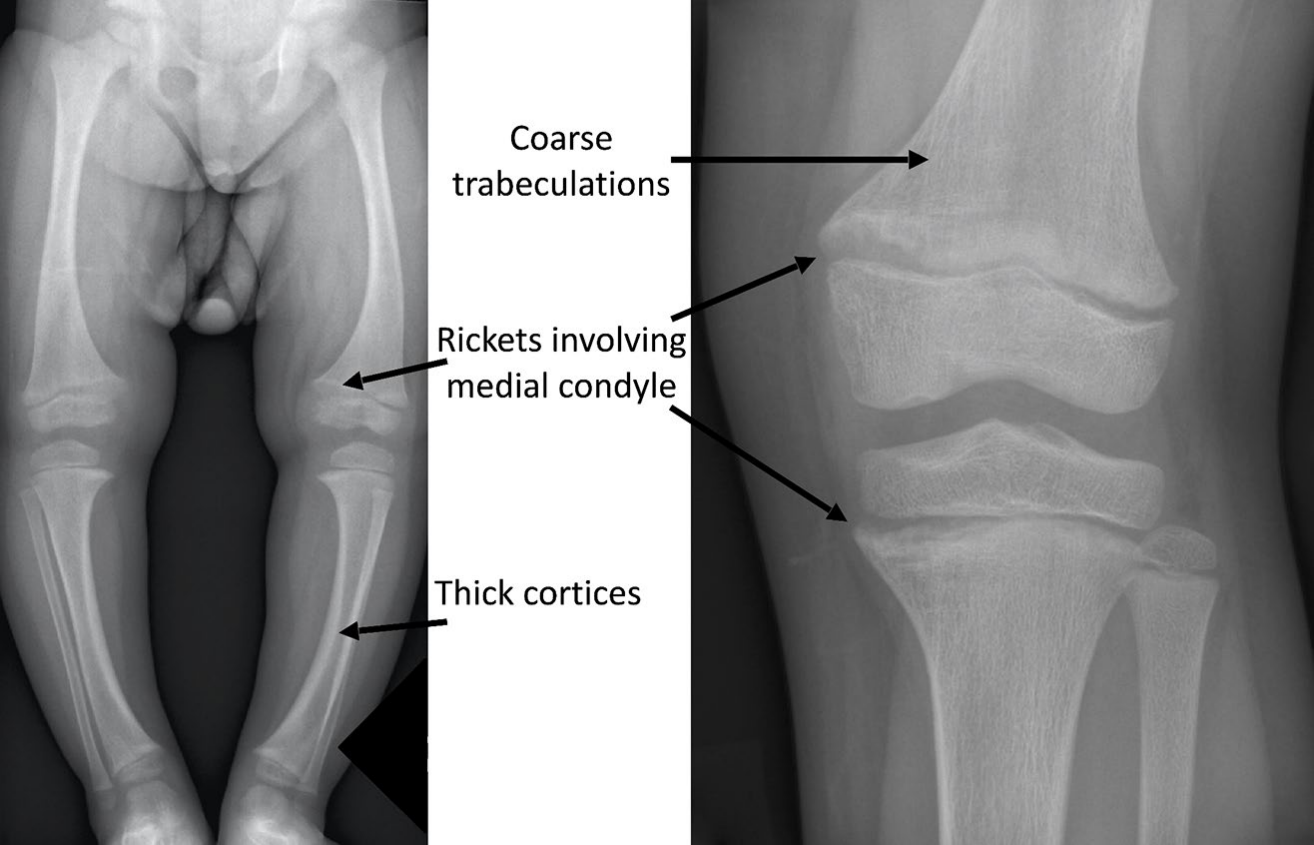
Radiological features of X-linked hypophosphatemic rickets. Lower limb and knee radiograph of a 5-y-old female showing characteristic changes of rickets affecting one of the femoral and tibial condyles, coarse trabeculations of the bones and thick cortices

Radiographs of children with high FGF23 hypophosphatemia are distinct from nutritional rickets (Fig. 1) with thicker cortices, coarse trabeculations, rickets affecting weight-bearing lower limb bones and either medial or lateral condyles more significantly affected than the other, medial in genu varum and lateral in genu valgum.

### Management of Hypophosphatemic Rickets

Management of FGF23-related hypophosphatemia (Table 2) is with oral phosphate supplements and active vitamin D analogues, alfacalcidol or calcitriol. Non-FGF23-related hypophosphatemia (Table 2) is managed with oral phosphate alone; active vitamin D analogues are *contraindicated* in these conditions. Additional solutes (potassium, bicarbonate and/or calcium) may be required in non-FGF23 related hypophosphatemia if there is proximal renal tubular acidosis with aminoaciduria.

Recent consensus guidance suggests using elemental phosphorus of 20–60 mg/kg body weight daily (0.7–2.0 mmol/kg daily) [8]. Since phosphorus is rapidly absorbed and excreted in the urine, 4–5 doses are required; doses >80 mg/kg/d are seldom required. Adjustment of the dose is based on serum alkaline phosphatase which is an important biomarker of the healing of rickets.

Administration of oral phosphorus reduces ionised serum calcium, increasing serum PTH which if not reduced can cause secondary and in some cases, tertiary hyperparathyroidism [9]. Active vitamin D analogues are therefore required to suppress PTH rise and also increase 1,25 (OH)_2_D which is low because of high FGF23. Active vitamin D analogues can be either calcitriol at an initial dose of 20–30 ng/kg/d in 2 divided doses or alfacalcidol of 30–50 ng/kg/d once a day [8]. The excess dose of calcitriol/alfacalcidol can cause increased excretion of calcium in the urine which in combination with excess urinary phosphate excretion can cause nephrocalcinosis. In some children, calcimimetic Cinacalcet may be required to prevent the rise in PTH while allowing oral phosphorus to heal rickets [10].

The outcome of medical management is variable as standard treatment with phosphorus and active vitamin D analogues still do not address the primary problem of excess FGF23 which continues to remain high [11]. Burosumab is a recombinant human monoclonal antibody which inhibits the activity of FGF23 [12]. In phase 3 clinical trial, Burosumab, compared to standard treatment, reduced urinary excretion of phosphate, normalised serum phosphate concentration and improved patient-reported outcome measures [13]. Treatment over a period of time heals rickets and normalises serum alkaline phosphatase. It is given as subcutaneous injections once every 2 wk and has been licensed in many countries for the management of XLH [14].

Iron deficiency state can increase FGF23 concentration and therefore in FGF23-related hypophosphatemic rickets, it can significantly worsen hypophosphatemic rickets [15]. Iron deficiency state especially in menstruating children and adolescent females should be therefore, adequately addressed.

There is evidence that early standard treatment reduces complications and improves height [16]. It is therefore crucial to make an early diagnosis by differentiating it from calcipenic rickets. Elevation of PTH differentiates between calcipenic and phosphopenic rickets and should be measured in initial screening blood tests for rickets.

## Vitamin D-Dependent Rickets (VDDR)

### Vitamin D Metabolism (Fig. 2)

**Fig. 2:**
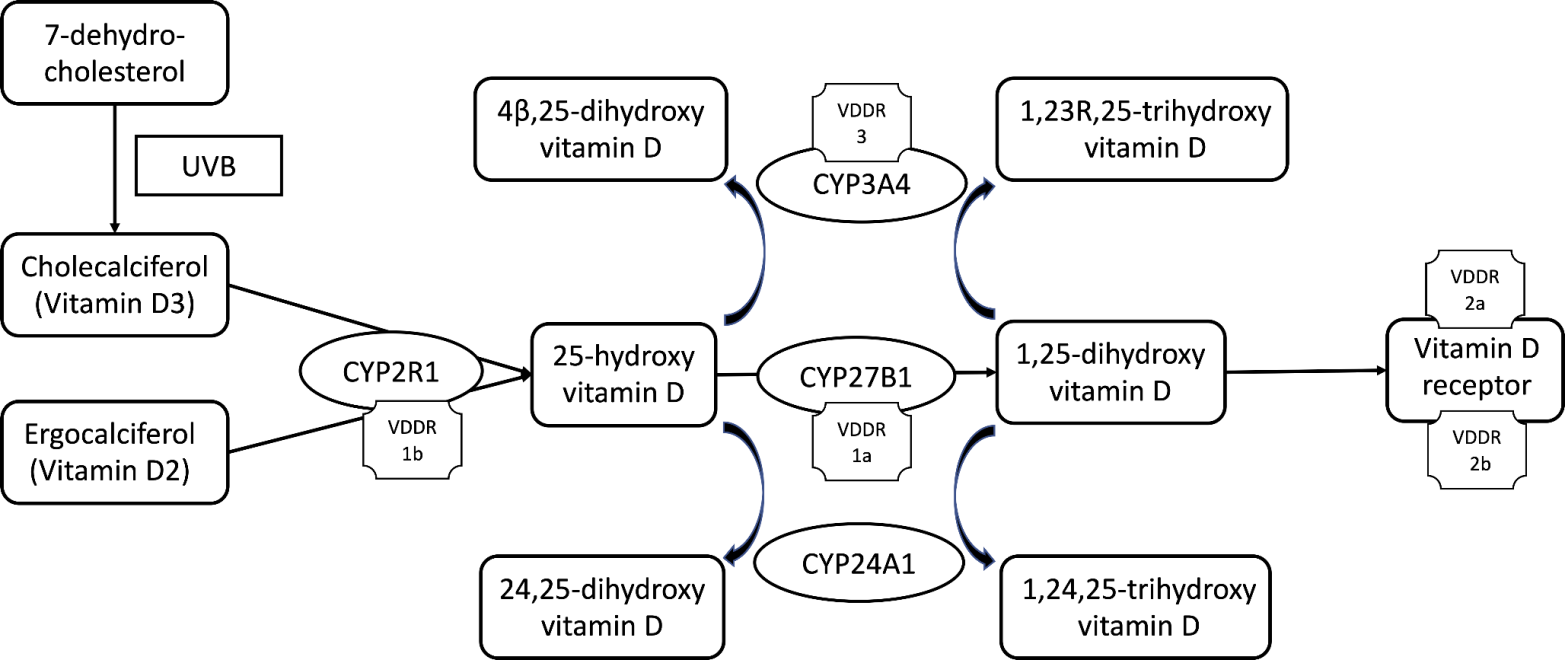
Vitamin D metabolism with defects related to vitamin D-dependent rickets. *VDDR* Vitamin D-dependent rickets

Vitamin D is a biologically inactive prohormone, that exists either as cholecalciferol (vitamin D3) or ergocalciferol (vitamin D2). Cholecalciferol is synthesised by ultraviolet (UVB) radiation of 7-dehydroxycholesterol present in the skin, whereas ergocalciferol is synthesised by fungi and yeast. Activation of these forms of vitamin D requires hydroxylation at two sites. 25-hydroxylation by 25-hydroxylase (*CYP2R1*) occurs in the liver, with the product 25-hydroxyvitamin D (25OHD) being the most abundant form of circulating vitamin D metabolite and the best indicator of body stores of vitamin D [17]. 1α-hydroxylation of 25OHD by 1α-hydroxylase (*CYP27B1*) then occurs in the kidney to produce the active metabolite 1,25-dihydroxy vitamin D [1,25(OH)_2_D]. The expression and activity of this enzyme is positively and negatively regulated by parathyroid hormone and FGF-23 respectively. 1,25(OH)_2_D binds to the nuclear vitamin D receptor (VDR) to exert its effects. In relation to rickets, the main effects of vitamin D are on the gut to stimulate calcium and phosphate absorption. 24-hydroxylation of 25OHD and 1,25(OH)_2_D by 24-hydroxylase (*CYP24A1*) in the kidney to 24,25-dihydroxyvitamin D and 1,24,25-trihydroxyvitamin D respectively results in inactivation of these products. However, a separate inactivation pathway for 25OHD and 1,25(OH)_2_D has more recently been established, by CYP3A4 in the liver [18].

### Types of Vitamin D-Dependent Rickets

VDDR refers to a group of conditions characterised by abnormalities in the pathway of vitamin D metabolism resulting in the reduced action of vitamin D (primarily intestinal calcium and phosphate absorption), which causes hypocalcemia, rickets and secondary hyperparathyroidism. Five types are recognised. Types of VDDR1 refer to abnormalities in producing active vitamin D, types of VDDR2 refer to abnormalities in VDR function and VDDR3 results in excessive inactivation of vitamin D. Table 3 summarises the features of each form of VDDR.

**Table 3:** Summary of genotype, biochemical differences and treatment options for each form of vitamin D-dependent rickets

	Inheritance	Gene	25OHD*	1,25(OH)_2_D	Treatment
VDDR1a (OMIM 264700)	AR	*CYP27B1*	Variable	↓ (occasionally normal)	Preferably calcitriol/alfacalcidol High doses of D2/D3 can be used instead Calcium supplementation
VDDR1b (OMIM 600081)	AR	*CYP2R1*	↓↓	Variable	Calcitriol of very high doses of D2/D3 More recently, calcifediol Calcium supplementation
VDDR2a (OMIM 277440)	AR	*VDR*	Normal/↑	↑↑	Very high doses of calcitriol/alfacalcidol High doses of calcium supplementation (may be required intravenously)
VDDR2b (OMIM 600785)	AR	Unknown	Normal/↑	↑↑	Very high doses of calcitriol/alfacalcidol High doses of calcium supplementation (may be required intravenously)
VDDR3 (OMIM 619073)	AD	*CYP3A4*	↓	↓	Very high doses of calcitriol/alfacalcidol or D2/D3 Calcium supplementation

*Can also be affected by vitamin D intake

*1,25(OH)*_*2*_*D* 1,25-dihydroxyvitamin D, *25OHD* 25-hydroxyvitamin D, *AD* Autosomal dominant, *AR* Autosomal recessive, *D2/D3* Ergocalciferol/Cholecalciferol, *VDDR* Vitamin D-dependent rickets

↓ Reduced, ↑ Elevated, ↓↓ Markedly reduced, ↑↑ Markedly elevated

#### VDDR1a

This condition is due to biallelic mutations in *CYP27B1* on chromosome 12q14.1. This results in an inability to 1α-hydroxylate 25OHD and subsequently an inability to produce active vitamin D. Some genotype-phenotype correlations are noted, depending on residual enzyme activity, although the majority of mutations result in complete loss of enzyme activity [19]. Children with this condition tend to present during infancy with hypocalcemic seizures, or in childhood with rickets, hypotonia and delayed motor development [20]. In the longer term, these children suffer from progressive skeletal deformities and short stature, if untreated. The typical biochemical findings are of hypocalcemia, hypophosphatemia, hyperphosphatasia, secondary hyperparathyroidism, and normal or elevated 25OHD with extremely low 1,25(OH)_2_D. However, an inappropriately normal 1,25(OH)_2_D has also been observed in genetically proven cases, which can cause diagnostic confusion [21]. Treatment is typically with therapeutic doses of active vitamin D [calcitriol; 1,25(OH)_2_D, twice daily] or active vitamin D analogues (alfacalcidol; 1α-hydroxyvitamin D once daily), which bypass the need for 1α-hydroxylation (Table 3). High concentrations of 25OHD can bind to the VDR directly, therefore high doses of cholecalciferol or ergocalciferol can be provided, but they carry a greater risk of hypercalcemia, hypercalciuria and nephrocalcinosis, which requires monitoring in the long-term regardless of treatment option used. Treatment quickly resolves the biochemical abnormalities and undermineralised skeleton. Improvements in stature take longer to occur, with more successful growth outcomes noted with earlier diagnosis and treatment [22]. Delayed diagnosis can occur not only with milder mutations but also because of misdiagnosis as nutritional rickets.

#### VDDR1b

This condition is similar to VDDR1a in clinical presentation but is due to mutations in *CYP2R1* (located at chromosome 11p15.2), which encodes for 25-hydroxylase [23]. Thus, the key biochemical difference between these two conditions is of very low 25OHD, which again can be confused with nutritional rickets. Interestingly, a gene dose effect is observed with this gene, whereby individuals with homozygous mutations have a more severe phenotype, undetectable  25OHD concentrations and almost no response to loading doses of vitamin D, whereas individuals carrying heterozygous mutations had a milder phenotype, only slightly reduced 25OHD concentrations and some response to loading doses of vitamin D [23]. Furthermore, the phenotype of these heterozygous individuals seems to improve with age [23]. Treatment is either with therapeutic doses of calcitriol (which bypasses 25-hydroxylation requirements) or with very high doses of cholecalciferol, along with calcium supplementation (Table 3). More recently, the development of calcifediol (25-hydroxycholecalciferol, once daily), has allowed a more rational treatment approach to this specific condition to be successfully undertaken [24].

#### VDDR2a

VDDR2a is due to biallelic mutations in *VDR*, the gene that encodes for the vitamin D receptor (VDR), located on chromosome 12q13.11. This results in resistance to 1,25(OH)_2_D. The vitamin D receptor is a nuclear receptor with an amino-terminal DNA-binding domain and a carboxy-terminal ligand-binding domain. When 1,25(OH)_2_D binds to the VDR, it heterodimerises with the retinoid-X receptor (RXR) before binding to vitamin D response elements (VDREs) in the promoter regions of vitamin D-regulated genes to initiate the transcription [25]. The clinical features and presentation of VDDR2a are similar to forms of VDDR1, except for the presence of partial or complete alopecia that develops within the first year of life. This alopecia is due to the role *VDR* plays in hair follicle cycling [26]. There appears to be a genotype-phenotype correlation in VDDR2a, whereby mutations affecting the ligand-binding domain tend to be present with a milder phenotype without alopecia, whereas mutations affecting the DNA-binding domain tend to be more severe, with poor response to therapy and the presence of alopecia [25]. Accordingly, alopecia can be used as a prognostic tool to predict severity, with the presence of alopecia associated with earlier diagnosis and greater treatment resistance [27]. The biochemistry of VDDR2a is similar to VDDR1a, except for elevated 1,25(OH)_2_D. Due to the resistance to 1,25(OH)_2_D, extremely high doses of active vitamin D or its analogues (preferably calcitriol) are required long-term, in addition to high doses of calcium supplementation (Table 3). In many cases, intravenous calcium supplementation through central venous access is required to achieve control of rickets and biochemical parameters. The response to treatment will be dependent upon residual VDR activity. Treatment will normalise biochemical parameters and will improve rickets with time, but the alopecia, if present, will persist. Interestingly, some cases have demonstrated an improvement with age, particularly following puberty, such that treatment requirements lessen [28]. This is thought to be due to the development of vitamin D-independent intestinal calcium absorption mechanisms with age [29].

#### VDDR2b

This condition has the same biochemical and clinical features as VDDR2a, except without a mutation in the *VDR* gene [30]. Therefore, the two conditions cannot be separated clinically, only by genetic testing. A specific genetic mutation has not yet been identified for VDDR2b. The function of VDR and the VDR-RXR heterodimer are retained, unlike VDDR2a, with the pathology thought to be due to the over-expression of heterogenous nuclear ribonucleoprotein C1/C2, which inhibits binding of the VDR-RXR heterodimer to VDREs [31, 32]. The treatment is identical to that for VDDR2a (Table 3).

#### VDDR3

This has only been recently described as a form of VDDR due to gain-of-function mutations in *CYP3A4* (located on chromosome 7q22.1), encoding a cytochrome P450 enzyme, resulting in increased inactivation of vitamin D metabolites [33]. Both cases described were short, with biochemical and radiological features of rickets. Both cases had detectable serum concentrations of vitamin D3, but low 25OHD and 1,25(OH)_2_D. These transiently increased with high doses of vitamin D3 or calcitriol, in keeping with the rapid degradation of vitamin D metabolites [33]. Thus, these cases will require lifelong treatment with high doses of cholecalciferol/ergocalciferol or active vitamin D or its analogues (Table 3). Interestingly, CYP3A4 has also been shown in in vitro studies to be the enzyme induced by P450-inducing drugs (such as rifampicin, carbamazepine and phenobarbital) to result in drug-induced osteomalacia [34].

### General Principles in the Management of VDDR

Although specific clinical, biochemical and treatment aspects have been referred to for each type of VDDR above, it is important to recognise some principles that are common to all forms. Calcium supplementation is often required, at the very least initially, in all forms of VDDR (Table 3). This is not only to address the presenting hypocalcemia but also to prevent further hypocalcemia that may occur due to the “hungry bones” during remineralisation upon commencing treatment. Secondary hyperparathyroidism is a universal feature of VDDR and will cause demineralisation of the bones. Adequate treatment of VDDR will hopefully resolve this in a timely manner, but the development of tertiary hyperparathyroidism needs to be monitored in cases of prolonged secondary hyperparathyroidism, with the use of cinacalcet considered in such cases [35]. Finally, regular biochemical monitoring is essential when treating all forms of VDDR. Under-treatment can be identified with ongoing hypocalcemia, hyperphosphatasia and secondary hyperparathyroidism, as well as with intermittent radiographs. As importantly, over-treatment must be avoided to prevent hypercalciuria and nephrocalcinosis with monitoring of urinary calcium-to-creatinine ratios undertaken to identify hypercalciuria.

## Conclusions

Although nutritional calcium and vitamin D deficiency is the commonest cause of rickets, rarer ‘refractory’ causes of rickets affecting phosphate and vitamin D metabolism, must be considered based particularly on specific biochemical findings, but also radiographic and clinical features, so that appropriate treatment and monitoring can be initiated in a timely manner.

## References

[1] Sabbagh Y, Carpenter TO, Demay MB (2005). Hypophosphatemia leads to rickets by impairing caspase-mediated apoptosis of hypertrophic chondrocytes. Proc Natl Acad Sci U S A.

[2] Perumal NL, Padidela R (2022). Phosphate homeostasis and disorders of phosphate metabolism. Curr Pediatr Rev.

[3] Padidela R, Nilsson O, Makitie O (2020). The international X-linked hypophosphataemia (XLH) registry (NCT03193476): rationale for and description of an international, observational study. Orphanet J Rare Dis.

[4] Lockitch G, Halstead AC, Albersheim S, MacCallum C, Quigley G (1988). Age- and sex-specific pediatric reference intervals for biochemistry analytes as measured with the Ektachem-700 analyzer. Clin Chem.

[5] Uday S, Shaw NJ, Mughal MZ (2021). Monitoring response to conventional treatment in children with XLH: value of ALP and Rickets Severity score (RSS) in a real world setting. Bone.

[6] Stark H, Eisenstein B, Tieder M, Rachmel A, Alpert G (1986). Direct measurement of TP/GFR: a simple and reliable parameter of renal phosphate handling. Nephron.

[7] Payne RB (1998). Renal tubular reabsorption of phosphate (TmP/GFR): indications and interpretation. Ann Clin Biochem.

[8] Haffner D, Emma F, Eastwood DM (2019). Clinical practice recommendations for the diagnosis and management of X-linked hypophosphataemia. Nat Rev Nephrol.

[9] Makitie O, Kooh SW, Sochett E (2003). Prolonged high-dose phosphate treatment: a risk factor for tertiary hyperparathyroidism in X-linked hypophosphatemic rickets. Clin Endocrinol (Oxf).

[10] Alon US, Levy-Olomucki R, Moore WV, Stubbs J, Liu S, Quarles LD (2008). Calcimimetics as an adjuvant treatment for familial hypophosphatemic rickets. Clin J Am Soc Nephrol.

[11] Imel EA, DiMeglio LA, Hui SL, Carpenter TO, Econs MJ (2010). Treatment of X-linked hypophosphatemia with calcitriol and phosphate increases circulating fibroblast growth factor 23 concentrations. J Clin Endocrinol Metab.

[12] Carpenter TO, Whyte MP, Imel EA (2018). Burosumab therapy in children with x-linked hypophosphatemia. N Engl J Med.

[13] Padidela R, Whyte MP, Glorieux FH (2021). Patient-reported outcomes from a randomized, active-controlled, open-label, phase 3 trial of burosumab versus conventional therapy in children with X-linked hypophosphatemia. Calcif Tissue Int.

[14] Padidela R, Cheung MS, Saraff V, Dharmaraj P (2020). Clinical guidelines for burosumab in the treatment of XLH in children and adolescents: british paediatric and adolescent bone group recommendations. Endocr Connect.

[15] Hogler W, Kapelari K (2020). Oral iron for prevention and treatment of rickets and osteomalacia in autosomal dominant hypophosphatemia. J Bone Miner Res.

[16] Makitie O, Doria A, Kooh SW, Cole WG, Daneman A, Sochett E (2003). Early treatment improves growth and biochemical and radiographic outcome in X-linked hypophosphatemic rickets. J Clin Endocrinol Metab.

[17] Khadilkar A, Kajale N, Oza C (2022). Vitamin D status and determinants in indian children and adolescents: a multicentre study. Sci Rep.

[18] Wang Z, Lin YS, Zheng XE (2012). An inducible cytochrome P450 3A4-dependent vitamin D catabolic pathway. Mol Pharmacol.

[19] Kaygusuz SB, Alavanda C, Kirkgoz T (2021). Does genotype-phenotype correlation exist in vitamin d-dependent rickets type IA: report of 13 new cases and review of the literature. Calcif Tissue Int.

[20] Dodamani MH, Sehemby M, Memon SS (2021). Genotype and phenotypic spectrum of vitamin D dependent rickets type 1A: our experience and systematic review. J Pediatr Endocrinol Metab.

[21] Dursun F, Ozgurhan G, Kirmizibekmez H, Keskin E, Hacihamdioglu B (2019). Genetic and clinical characteristics of patients with vitamin d dependent rickets type 1A. J Clin Res Pediatr Endocrinol.

[22] Edouard T, Alos N, Chabot G, Roughley P, Glorieux FH, Rauch F (2011). Short- and long-term outcome of patients with pseudo-vitamin D deficiency rickets treated with calcitriol. J Clin Endocrinol Metab.

[23] Thacher TD, Fischer PR, Singh RJ, Roizen J, Levine MA (2015). CYP2R1 mutations impair generation of 25-hydroxyvitamin d and cause an atypical form of vitamin D deficiency. J Clin Endocrinol Metab.

[24] Molin A, Wiedemann A, Demers N (2017). Vitamin D-dependent rickets type 1B (25-hydroxylase deficiency): a rare condition or a misdiagnosed condition?. J Bone Miner Res.

[25] Malloy PJ, Pike JW, Feldman D (1999). The vitamin D receptor and the syndrome of hereditary 1,25-dihydroxyvitamin D-resistant rickets. Endocr Rev.

[26] Bikle DD, Elalieh H, Chang S, Xie Z, Sundberg JP (2006). Development and progression of alopecia in the vitamin D receptor null mouse. J Cell Physiol.

[27] Marx SJ, Bliziotes MM, Nanes M (1986). Analysis of the relation between alopecia and resistance to 1,25-dihydroxyvitamin D. Clin Endocrinol (Oxf).

[28] Nicolaidou P, Tsitsika A, Papadimitriou A (2007). Hereditary vitamin D-resistant rickets in greek children: genotype, phenotype, and long-term response to treatment. J Pediatr Endocrinol Metab.

[29] Tiosano D, Hadad S, Chen Z (2011). Calcium absorption, kinetics, bone density, and bone structure in patients with hereditary vitamin D-resistant rickets. J Clin Endocrinol Metab.

[30] Giraldo A, Pino W, Garcia-Ramirez LF, Pineda M, Iglesias A (1995). Vitamin D dependent rickets type II and normal vitamin D receptor cDNA sequence. A cluster in a rural area of Cauca, Colombia, with more than 200 affected children. Clin Genet.

[31] Chen H, Hewison M, Adams JS (2006). Functional characterization of heterogeneous nuclear ribonuclear protein C1/C2 in vitamin D resistance: a novel response element-binding protein. J Biol Chem.

[32] Chen H, Hewison M, Hu B, Adams JS (2003). Heterogeneous nuclear ribonucleoprotein (hnRNP) binding to hormone response elements: a cause of vitamin D resistance. Proc Natl Acad Sci U S A.

[33] Roizen JD, Li D, O’Lear L (2018). CYP3A4 mutation causes vitamin D-dependent rickets type 3. J Clin Invest.

[34] Wang Z, Lin YS, Dickmann LJ (2013). Enhancement of hepatic 4-hydroxylation of 25-hydroxyvitamin D3 through CYP3A4 induction in vitro and in vivo: implications for drug-induced osteomalacia. J Bone Miner Res.

[35] Nicolescu RC, Lombet J, Cavalier E (2018). Vitamin D-resistant rickets and cinacalcet-one more favorable experience. Front Pediatr.

